# Effects of Intercropping *Morchella* Between Apple Tree Rows on Soil Physicochemical Properties and Tree Performance

**DOI:** 10.3390/biology15151303

**Published:** 2026-08-05

**Authors:** Xiaodan Wang, Liuyuan Bao, Chengcui Yang, Li Dong, Zhongyan Tang, Jie Luo, Fajun Xiang, Huan Qin, Yonghong He, Shunqiang Yang

**Affiliations:** 1 College of Plant Protection, Yunnan Agricultural University, Kunming 650201, China; wxd18787100724@163.com (X.W.); 14769158780@163.com (L.D.); 2 Key Laboratory of Edible Fungi Resources Innovation Utilization and Cultivation, Zhaotong University, Zhaotong 657000, China; 47015@ztu.edu.cn (L.B.); ycjgxcm@163.com (C.Y.); 13988395247@nwsuaf.edu.cn (Z.T.); 19508708280@163.com (J.L.); m19869270608@163.com (F.X.); 13408877607@163.com (H.Q.); 3 Yunnan Key Laboratory of Smart Villages and Agri-Cultural-Tourism Integration, Zhaotong University, Zhaotong 657000, China; 4 College of Agriculture and Life Sciences, Zhaotong University, Zhaotong 657000, China

**Keywords:** *Morchella*, *Malus domestica*, fruit quality, soil nutrient dynamics, soil enzyme activity, photosynthetic physiology

## Abstract

To investigate the effects of interplanting *Morchella* (morel mushrooms) between rows of apple trees on orchard soil properties and apple tree performance, a field experiment was conducted using a cultivation system combining small-arch tunnels with plastic film covering. The *Morchella* was interplanted in a five-year-old apple orchard, with plots without *Morchella* cultivation serving as the control. Measurements included *Morchella* yield and quality, apple fruit quality, and leaf photosynthetic performance. Additionally, soil physicochemical properties and enzyme activities were analyzed across the 0–40 cm soil layer at different depths. The results demonstrated that: (1) Intercropping *Morchella* in apple interrows proved to be agronomically feasible, yielding a fresh mushroom production of 1333.74 g/m^2^. Moreover, this cultivation system significantly enhanced the nutritional quality of the harvested morels, as evidenced by marked increases in crude fiber, total sugars, reducing sugars, and free amino acid contents. (2) In the 0–20 cm soil layer, the *Morchella* cultivated plots exhibited significantly higher natural water content compared to the control. The measured values for soil pH, alkali-hydrolyzable nitrogen, organic carbon, catalase activity, and sucrase activity were 5.46, 6.73 mg/kg, 38.30 g/kg, 411.86 μ mol/h/g, and 9.89 mg/d/g, respectively, all significantly greater than those in the control (*p* < 0.05). In the 20–40 cm layer, however, soil available potassium and organic carbon contents were 418.37 mg/kg and 28.45 g/kg, respectively, both significantly lower than the control (*p* < 0.05). Across both treatments, the values of soil pH, alkali -hydrolyzable nitrogen, organic carbon, available phosphorus, available potassium, and the activities of urease, amylase, catalase, and sucrase generally decreased with increasing soil depth. Notably, in the non-cultivated control plots, the contents of alkali-hydrolyzable nitrogen and organic carbon increased with depth. (3) Interplanting *Morchella* improved apple fruit quality to a certain extent, significantly increasing individual fruit fresh weight, fruit shape index, and the contents of reducing sugars, total sugars, and soluble solids. (4) Interplanting *Morchella* also enhanced the photosynthetic rate of apple trees to some degree, significantly increasing transpiration rate, intercellular CO_2_ concentration, and stomatal conductance. Collectively, interplanting *Morchella* in apple orchards can produce a reasonably high yield of high-quality mushrooms, increase individual apple fruit fresh weight, improve the physicochemical properties of the 0–20 cm soil layer, and enhance the photosynthetic performance of the apple trees. These combined benefits demonstrate clear practical potential and support the promotion and application of this intercropping system in orchard production.

## Simple Summary

Growing morel mushrooms alongside apple trees may offer multiple benefits for both farmers and the land. In our study, we planted *Morchella* between rows of young apple trees and observed the results. The mushrooms produced a plentiful harvest and were rich in nutrients. We found that morel cultivation improved the surface soil by retaining more water and increasing natural nutrients and enzyme activity. While some nutrient levels changed in deeper soil layers, the overall soil condition stayed healthy. Apple trees grown next to morels produced larger, sweeter, and more shapely fruit. These trees also showed better photosynthesis, indicating more efficient growth. Overall, interplanting morels in apple orchards can enhance soil health and fruit quality while giving farmers an extra valuable crop to sell.

## 1. Introduction

*Morchella esculenta* (L.) Pers., commonly known as morel mushroom, yellow morel, or sponge mushroom, is an edible and medicinal fungus belonging to the genus *Morchella* within the family Morchellaceae, order Pezizales, class Pezizomycetes, and subphylum Ascomycotina [1,2]. As a prized and rare edible mushroom, *esculenta* is highly sought after in commercial markets. It is rich in protein, amino acids, vitamins, minerals, and bioactive compounds, including organic germanium. The species exhibits a broad spectrum of health-promoting properties, including antitumor, immunomodulatory, antibacterial, anti-fatigue, antioxidant, hypolipidemic, and hepatoprotective effects. Consequently, it holds considerable economic importance and substantial scientific research value [3,4].

In recent years, the introduction of nutrient bag technology has accelerated the expansion of *Morchella* cultivation. Consequently, cultivation systems have diversified considerably, evolving from the original flat shed method to include understory cultivation, facility-based production, mechanized farming on plains, and temperature-controlled greenhouse systems [5]. Among these, understory cultivation has emerged as one of the primary models for extension and application, owing to its efficient utilization of forest land resources, enhancement of under-canopy economic returns, and reduction in production costs [6]. Previous studies have reported that understory intercropping with edible fungi can improve soil physicochemical properties while simultaneously increasing crop yield and quality. For instance, interplanting *Stropharia rugosoannulata* in vineyards has been reported to increase soil organic matter, hydrolyzable nitrogen, and available potassium, while also significantly improving grape quality [7]. Further studies on cultivating *S. rugosoannulata* and *Dictyophora echinovolvata* under grapevines demonstrated that soil water content, pH, organic matter, hydrolyzable nitrogen, and available potassium were all higher than those of the control, and also enhanced grape yield and quality [8]. Interplanting *S. rugosoannulata* in young tea plantations in winter was found to improve soil physicochemical properties, protect young tea plants from winter damage, and boost spring tea yield [9]. The return of substrate and spent mushroom residue to the field after understory *Morchella* cultivation may also influence soil nutrient dynamics. A study investigating the application of varying amounts of spent *Morchella sextelata* substrate in a *Phyllostachys edulis* forest showed that total phosphorus (TP) content in the topsoil increased by 12–20 times, while available phosphorus (AP) and available potassium (AK) also increased significantly; urease (UA) and sucrase (SA) activities were enhanced by 10–17 times and 3–5 times, respectively; however, catalase (CAT) and acid phosphatase (ACP) activities were temporarily inhibited [10]. Research on rotating *Morchella* with flue-cured tobacco and incorporating spent substrate into the field showed that a tobacco-*Morchella*-tobacco rotation system with rotary tillage improved soil aeration and aggregation, boosted fertility and microbial abundance, promoted leaf growth, increased tobacco yield and value, and improved leaf chemical balance [11]. Understory cultivation promotes plant growth by improving soil physicochemical properties. By ameliorating soil physicochemical properties, understory cultivation of edible fungi indirectly promotes plant growth. A study on the effects of *Grifola frondosa* residue returning on the growth and photosynthetic traits of chestnut seedlings revealed that this practice increased leaf area, specific leaf area, and leaf SPAD values [12]. Notably, the net photosynthetic rate (Pn) of chestnut seedlings increased by up to 25.92%, accompanied by concurrent elevations in stomatal conductance (Gs) and intercellular CO_2_ concentration (Ci), indicating a marked enhancement of photosynthetic performance. Related research has demonstrated that understory cultivation significantly upregulates the expression of genes involved in the photosynthetic pathway of *Coptis chinensis*, while concurrently increasing chlorophyll content and plant fresh weight, thereby confirming the ameliorative effect of the understory environment on photosynthetic capacity [13]. In summary, understory cultivation of edible fungi not only makes efficient use of forest land resources, enhances the yield and quality of the edible fungi, and achieves a dual harvest of both the main crop and the fungi, but also improves soil physicochemical properties to a certain extent and promotes the growth and development of the fruit trees.

Currently, precise statistics on the total area of understory edible fungi cultivation in China are unavailable, but national targets (reaching 8 million mu by 2030) and local practices have clearly demonstrated its enormous development potential. Integrating edible fungi cultivation with orchards and forestry offers a practical and viable pathway to address the “impossible triangle” of ensuring food security, mitigating climate change, and conserving biodiversity. Currently, understory cultivation of edible fungi has been widely implemented across multiple regions and has achieved notable economic benefits. Zhaotong City in Yunnan Province is a major apple-producing area in the cool, high-altitude region of southwestern China. By the end of 2024, the apple cultivation area in Zhaotong had reached approximately 56,667 hectares, providing abundant understory space resources [14,15]. Leveraging the favorable conditions of apple orchards—such as suitable canopy closure, high air humidity, appropriate light intensity, ample oxygen availability, and relatively low diurnal temperature fluctuations—the cultivation of *Morchella* beneath apple trees represents a promising model for achieving synergistic gains in both ecological conservation and economic development. To date, studies on the effects of understory *Morchella* cultivation in apple orchards on soil physicochemical properties and tree performance remain scarce. To address this gap, the present study was conducted by interplanting *Morchella* between apple tree rows, with the aim of evaluating its effects on *Morchella* yield and quality, apple fruit quality, soil physicochemical properties, and tree growth and development. This study is intended to provide preliminary baseline data and a scientific reference for understory *Morchella* cultivation in orchard systems.

## 2. Materials and Methods

### 2.1. Site Description

The field experiment was conducted from December 2024 to September 2025 at the apple base of Haoran Agricultural Development Co., Ltd., located in Sujiayuan Town, Zhaoyang District, Zhaotong City, Yunnan Province (103°75″ E, 27°36″ N; elevation 1915 m). The study site is characterized by a mean annual temperature of 11 °C, an average annual precipitation of 750 mm, and a frost-free period of 230 days [16]. The soil was classified as brown earth, with a pH of 5.25, a field water-holding capacity of 38.06%, and a bulk density of 1.09 g/cm^3^. Alkali-hydrolyzable nitrogen measured 5.67 mg/kg, organic matter content was 34.57 g/kg, and available phosphorus and potassium were 5.13 mg/kg and 407.28 mg/kg, respectively.

### 2.2. Test Materials

The *Morchella* spawn and nutrient packs were both purchased from Zhaotong Longxing Biotechnology Co., Ltd. Ludian County, Zhaotong City, Yunnan Province, China (Table 1). 

### 2.3. Experimental Design

Five-year-old ‘Red Fuji’ apple trees were selected as the experimental subjects. On 24 November 2024, *Morchella* spawn was inoculated under a single-factor randomized complete block design with two treatments: Treatment A (intercropping with *Morchella*) and CK (non-intercropping control). Each treatment was replicated three times, with individual tree rows serving as the replicate units. For Treatment A, raised beds were established along the longitudinal axis between the tree rows for mushroom cultivation. Prior to sowing, the soil was rotary-tilled to a depth of 10–15 cm, with clods broken up and the surface leveled by raking, followed by thorough irrigation to saturate the subsoil. Sunken planting beds (10 m × 1 m × 0.10 m in length × width × depth) were then prepared by trenching to facilitate drainage and moisture retention. On each planting bed, three parallel furrows (approximately 5 cm deep and 10 cm wide) were opened at 20 cm intervals. The *Morchella* spawn was crumbled, mixed with an appropriate amount of potassium dihydrogen phosphate, and then evenly broadcast into the furrows, with each row receiving approximately five spawn bags (equivalent to about 5601.7 g per row). After sowing, the furrows were covered with a 3–5 cm layer of soil, followed by a layer of black plastic mulch. Subsequently, low tunnels were erected and covered with transparent film and shade netting to regulate temperature, humidity, and light conditions. Upon observation of mycelial growth with the formation of mycelial frost (primordia initials), nutrient supplement bags were placed on the soil surface at 30 cm intervals, with 120 bags deployed per row. Throughout the growing season, standard *Morchella* cultivation practices were followed for water management, environmental regulation, and pest and disease control. For the CK treatment, no *Morchella* spawn was sown, and neither nutrient supplement bags nor plastic mulch were applied. All rows, regardless of treatment (A and CK), received identical orchard management practices throughout the experimental period.

The open-field conventional cultivation treatment (L) was established on a flat, open site. The planting method, sowing rate, nutrient bag application, and field management practices were identical to those of Treatment A, including furrow sowing, soil coverage, plastic mulching, and the installation of low tunnels covered with transparent film and shade netting. However, this treatment was not placed under the apple tree canopy and was therefore not subject to root competition or canopy shading from the fruit trees, thereby representing the standard open-field Morchella cultivation practice in the region.

Throughout the experimental period, all apple trees received uniform agronomic management. Irrigation was applied as needed based on soil moisture conditions. A standardized fertilization regime was adopted across all plots, with no differences among treatments. Pest and disease control measures were implemented uniformly across the entire orchard to minimize confounding effects. Manual weeding was carried out as required to maintain consistent growing conditions. All management practices were executed identically for both the intercropping and control treatments, ensuring that the sole difference between treatments was the presence or absence of *Morchella* cultivation.

### 2.4. Sample Collection and Preparation

*Morchella* collection: From March to April 2025, morel mushrooms were harvested, and the yield was measured. The treatment group cultivated under the apple canopy was designated as P- YDJ, while the open field cultivated control group was designated as L-YDJ. Each sampling plot measured 2 m^2^, and each treatment was replicated three times. Fresh weight was determined immediately after harvest to calculate yield. The harvested morels were dried in an oven at 50 °C to constant weight, then ground to pass through a 60 mesh sieve. The resulting powder was sealed and stored for subsequent analyses.

Soil sampling: In May 2025, soil sampling was conducted in each treatment plot. Within the interrows of the apple trees, five sampling points were evenly distributed along the longitudinal axis of the planting bed, while avoiding irrigation points, the edges of the plastic mulch, and the areas outside the canopy drip line. Soil samples were collected from two depths at each point: 0–20 cm (designated as A_0_) and 20–40 cm (designated as A_1_). The five subsamples collected from the same plot and the same soil depth were thoroughly mixed to form a composite sample. For the 0–20 cm layer, fresh soil was carefully cleared of stones and plant debris and then used for the determination of soil physical properties (e.g., bulk density and water content). An additional portion of each composite sample was air-dried, passed through a 100-mesh sieve, and sealed for storage prior to analysis of soil nutrient contents and enzyme activities. Using the same procedure, soil samples were also collected from the interrows of apple trees without *Morchella* cultivation, at depths of 0–20 cm (designated CK_0_) and 20–40 cm (designated CK_1_), to serve as controls.

Apple sampling: Apple yield was assessed on 17 November 2025. Five apple trees from the *Morchella*-cultivated plots (designated PG-A) and five from the non-cultivated control plots (designated PG-CK) were selected, ensuring that all trees were of the same variety and exhibited comparable growth vigor. From each tree, ten apples of similar maturity were harvested from the four cardinal directions (east, south, west, and north) and pooled to form a composite sample. Apples harvested from trees without *Morchella* intercropping served as the control. Upon collection, the apples were stored at 4 °C prior to the assessment of fruit quality parameters.

### 2.5. Measurement of Parameters

Quality assessment of *Morchella* and apple fruits: For morel fruiting bodies, ash content was determined following GB/T 6438-2007 [17], crude fat content following GB/T 6433-2006 [18], and crude fiber content following GB/T 5009.10-2003 [19]. Reducing sugar content was determined by the 3,5-dinitrosalicylic acid (DNS) method [20]. Crude protein content was assayed using the Coomassie Brilliant Blue G-250 staining method [21], and vitamin C content was measured by the 2,6-dichlorophenolindophenol titration method [22]. Total sugar content was measured using the anthrone colorimetric method. Free amino acid content was determined using the ninhydrin colorimetric method. For apple fruits, titratable acidity was measured by NaOH titration, soluble solids content was determined using a handheld refractometer, and fruit firmness was measured with a GY-4 digital fruit hardness tester, Shanghai Baosheng Industrial Development Co., Ltd. (BosinTech), Shanghai, China.

Soil property measurements: Field water capacity, bulk density, total porosity, capillary porosity, and non-capillary porosity were determined following the methods described by Wang et al. [23]. Soil pH, alkali-hydrolyzable nitrogen, available phosphorus, and available potassium were measured according to the protocols outlined in Soil Agrochemistry Analysis [24]. Soil organic carbon content was determined using a Multi N/C 3100 analyzer, Analytik Jena AG (Analytik Jena GmbH+Co. KG), Jena, Germany. Soil enzyme activities—including catalase, sucrase, amylase, and urease—were assayed using commercial soil enzyme assay kits (purchased from Suzhou Grace Biotechnology Co., Ltd., Suzhou, Jiangsu, China).

Photosynthetic measurements: Measurements were conducted on clear days in June, July, August, and September 2025, between 10:30 and 11:30 a.m., using a Li 6800 portable photosynthesis system, LI-COR Biosciences (LI-COR, Inc.), Lincoln, NE, USA. Apple trees of the same variety and comparable growth vigor, located in the central area of the orchard, were selected from both the *Morchella* cultivated and non-cultivated plots. Functional leaves from new shoots at the same height on the southern side of the canopy were chosen for measurement. The *Morchella* cultivated trees were designated A_6_, A_7_, A_8_, and A_9_ (for June through September, respectively), while the corresponding control trees were designated CK_6_, CK_7_, CK_8_, and CK_9_. Li-6800-01-A red- blue light source was used, with photosynthetically active radiation (PAR) set to 1500 μmol/(m^2^·s), leaf chamber temperature maintained at 25 °C, ambient CO_2_ concentration at 400 μmol/mol (corrected from the original value), and relative humidity at 50%. The photosynthetic parameters measured included net photosynthetic rate [Pn, μmol/(m^2^·s)], transpiration rate [Tr, mmol/(m^2^·s)], stomatal conductance [Gs, mmol/(m^2^·s)], and intercellular CO_2_ concentration [Ci, μmol/mol].

### 2.6. Data Processing

Data were organized using Microsoft Excel 2019. Statistical analyses were performed with DPS 9.01 and significant differences among treatments were determined using the least significant difference (LSD) test. Graphical representations were generated using OriginPro 2022.

## 3. Results

### 3.1. Effects of Different Cultivation Sites on Morel Yield and Quality

The yield and drying ratio of *Morchella* varied between cultivation sites (Table 2). Understory cultivation in the apple orchard yielded 1333.74 g/m^2^ of fresh mushrooms, which was 16.11% higher than that of open-field cultivation, though the difference was not statistically significant. The drying ratio under the apple canopy was 9.71%, representing a significant reduction of 3.34% compared to the open-field control. This indicates that intercropping *Morchella* under apple trees can achieve a measurable yield.

The nutritional composition of *Morchella* fruiting bodies differed significantly between cultivation sites (Table 3). Understory cultivation in the apple orchard resulted in markedly higher contents of crude fiber (14.54%), total sugars (19.38%), reducing sugars (27.65%), and free amino acids (0.78%) compared to the control, with all differences being statistically significant. Conversely, the contents of ash (7.53%), crude protein (6.26%), and vitamin C (0.16%) were significantly lower than those in the control. Crude fat content was also higher under the apple canopy, though the difference did not reach statistical significance. In summary, the effects of understory *Morchella* cultivation in apple orchards on mushroom quality varied across different nutritional indices. Understory cultivation significantly increased the contents of crude fiber, total sugar, reducing sugar, and free amino acids, while simultaneously leading to marked reductions in ash, crude protein, and vitamin C contents. Collectively, these findings suggest that apple-based intercropping can partially improve the overall quality of *Morchella*.

### 3.2. Effects of Morel Cultivation Between Apple Rows on the Soil Environment Under Apple Orchards

#### 3.2.1. Effects of Different Treatments on the Physical Properties of Soil Between Apple Rows

Intercropping *Morchella* between apple rows affected various soil physical properties (Table 4). Specifically, natural water content, field water capacity, total porosity, capillary porosity, and non-capillary porosity were all higher in the treated plots than in the control. Among these, natural water content (21.45%) was significantly greater than that of the control. Bulk density in the treated plots (1.51 g/cm^3^) was lower than in the control, though the difference was not statistically significant.

#### 3.2.2. Effects of Different Treatments on the Chemical Properties of the Soil Between Apple Rows

Intercropping *Morchella* between apple rows influenced soil chemical properties (Table 5). In the 0–20 cm soil layer, the *Morchella*-cultivated plots exhibited significantly higher pH (5.46), alkali-hydrolyzable nitrogen (6.73 mg/kg), and organic carbon (38.30 g/kg) compared to the control (*p* < 0.05). Although available phosphorus and available potassium contents were lower than those in the control, the differences were not statistically significant. In the 20–40 cm layer, soil available potassium (418.37 mg/kg) and organic carbon (28.45 g/kg) were both significantly lower than those in the control (*p* < 0.05). Soil pH and available phosphorus were higher than in the control, while alkali-hydrolyzable nitrogen was lower; however, none of these differences reached statistical significance.

#### 3.2.3. Effects of Different Treatments on Soil Chemical Properties Across Different Soil Layers in the Apple Interrows

**Figure 1:**
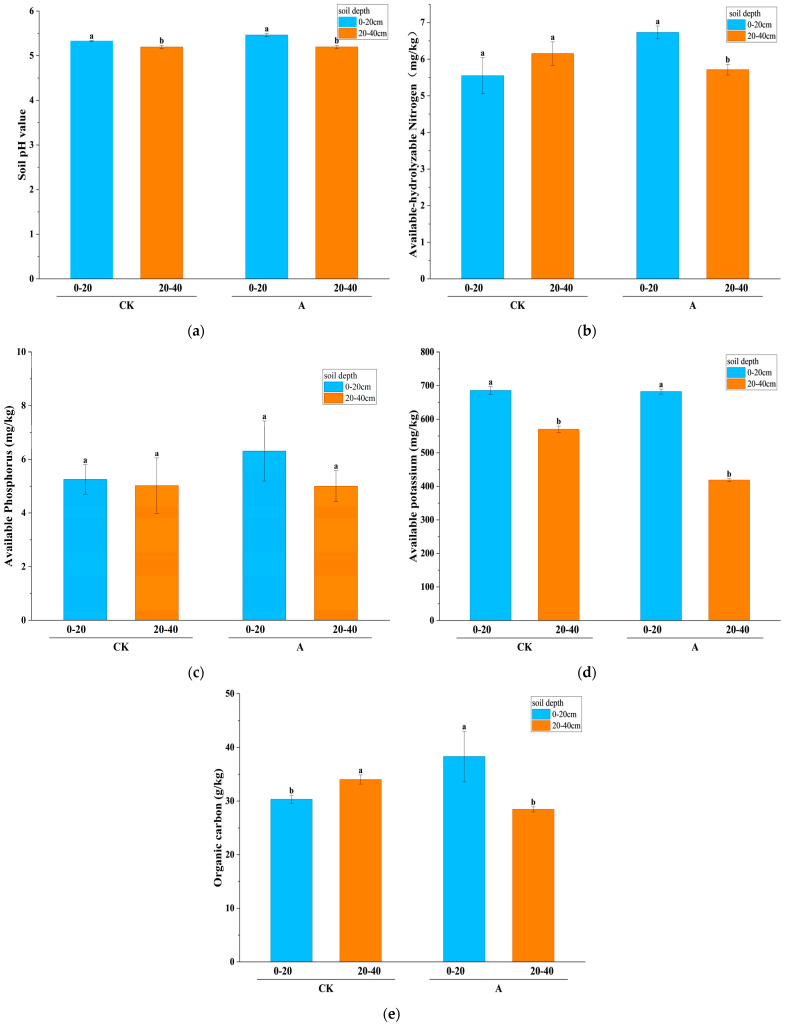
Effects of different treatments on the physicochemical properties of soil across different soil layers in the apple interrows. Different lowercase letters in the same column indicate significant differences among treatments at *p* < 0.05. (**a**) Soil pH value; (**b**) alkali-hydrolyzable nitrogen; (**c**) available phosphorus; (**d**) available potassium; (**e**) organic carbon.

The chemical properties of soils in the *Morchella* cultivated and non-cultivated plots differed between soil layers (Figure 1). In the *Morchella*-cultivated plots, the contents of pH, alkali-hydrolyzable nitrogen, available phosphorus, available potassium, and organic carbon all decreased with increasing soil depth, with reductions of 4.76%, 15.13%, 3.34%, 38.69%, and 25.72%, respectively. Among these, the decreases in pH, alkali-hydrolyzable nitrogen, available potassium, and organic carbon were statistically significant, whereas the decrease in available phosphorus was not. In the non-cultivated control plots, pH, available phosphorus, and available potassium decreased with increasing soil depth by 2.50%, 20.63%, and 16.85%, respectively. The decreases in pH and available potassium were statistically significant, while the decrease in available phosphorus was not. In contrast, alkali-hydrolyzable nitrogen and organic carbon in the control plots increased with depth by 10.89% and 12.14%, respectively, with the increase in organic carbon being statistically significant and that in alkali-hydrolyzable nitrogen being non-significant.

#### 3.2.4. Effects of Different Treatments on Soil Enzyme Activities in the Apple Interrows

Intercropping *Morchella* between apple rows influenced soil enzyme activities (Table 6). In the 0–20 cm soil layer, the *Morchella*-cultivated plots exhibited catalase and sucrase activities of 411.86 μmol/h/g and 9.89 mg/d/g, respectively, both of which were significantly higher than those in the control (*p* < 0.05). Urease activity was higher than in the control, while amylase activity was lower, though neither difference reached statistical significance. In the 20–40 cm layer, amylase and sucrase activities were higher than those in the control, whereas urease and catalase activities were lower. However, none of these differences were statistically significant.

#### 3.2.5. Effects of Different Treatments on Soil Enzyme Activities Across Different Soil Layers in the Apple Interrows

**Figure 2:**
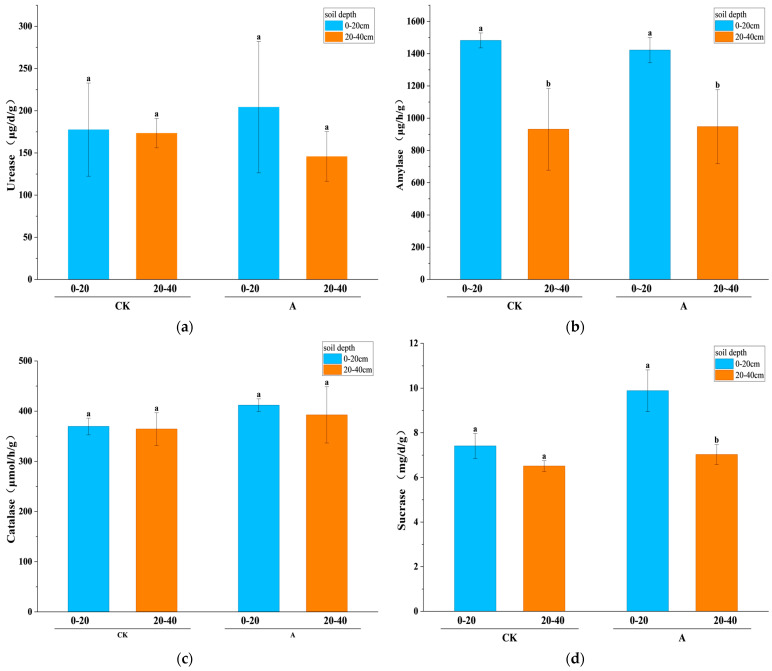
Effects of different treatments on soil enzyme activities across different soil layers in the apple interrows. Different lowercase letters in the same column indicate significant differences among treatments at *p* < 0.05 (**a**) Urease; (**b**) amylase; (**c**) catalase; (**d**) sucrase.

Soil enzyme activities in the *Morchella*-cultivated and non-cultivated plots differed between soil layers (Figure 2). In the *Morchella*-cultivated plots, the activities of urease, amylase, catalase, and sucrase all decreased with increasing soil depth, with reductions of 28.63%, 33.37%, 4.68%, and 28.91%, respectively. Among these, the decreases in amylase and sucrase activities were statistically significant, whereas those in urease and catalase activities were not. In the non-cultivated control plots, urease, amylase, catalase, and sucrase activities decreased with depth by 2.34%, 37.15%, 1.40%, and 12.14%, respectively. Among these, the decrease in sucrase activity was statistically significant, while the changes in urease, catalase, and amylase activities were not.

### 3.3. Effects of Different Treatments on Apple Yield and Quality

Intercropping *Morchella* between apple rows significantly influenced the nutritional composition of apple fruits (Table 7). In the *Morchella*-cultivated plots, both individual fresh weight and fruit shape index were significantly higher than those in the control (*p* < 0.05). Regarding internal fruit quality parameters, reducing sugars (82.53 mg/g), total sugars (101.71 mg/g), and soluble solids (15.81%) were all significantly elevated compared to the control (*p* < 0.05). Titratable acidity and vitamin C content were lower than those in the control, while fruit firmness was higher; however, none of these differences reached statistical significance.

### 3.4. Effects of Different Treatments on Photosynthetic Performance of Apple Trees

Different treatments exerted notable effects on the photosynthetic performance of apple trees (Table 8). In the *Morchella*-cultivated plots, the net photosynthetic rate (Pn) of apple trees increased by 4.76% and 11.84% in June and July, respectively, compared to the control (CK), whereas it decreased by 7.45% and 5.02% in August and September, respectively. However, none of these differences reached statistical significance. The transpiration rate (Tr) of apple trees was consistently higher in the *Morchella*-cultivated plots across all four months, with increases of 16.67%, 25.00%, 9.09%, and 16.67% in June, July, August, and September, respectively, compared to CK. The increases in July, August, and September were statistically significant. The intercellular CO_2_ concentration (Ci) in apple leaves was also elevated in the treated plots, with increases of 0.42%, 4.45%, 1.51%, and 6.76% in June, July, August, and September, respectively, relative to CK. The increase observed in September reached statistical significance. Stomatal conductance (Gs) of apple trees was consistently higher in the treatment group across all months, with increases of 22.30%, 25.79%, 6.15%, and 14.33% in June, July, August, and September, respectively, compared to CK. The increase in July was statistically significant. In summary, compared with the CK treatment, intercropping with *Morchella* significantly enhanced the transpiration rate, intercellular CO_2_ concentration, and stomatal conductance of apple trees.

### 3.5. Effects of Different Treatments on Photosynthesis in Apple Trees in Different Months

**Figure 3:**
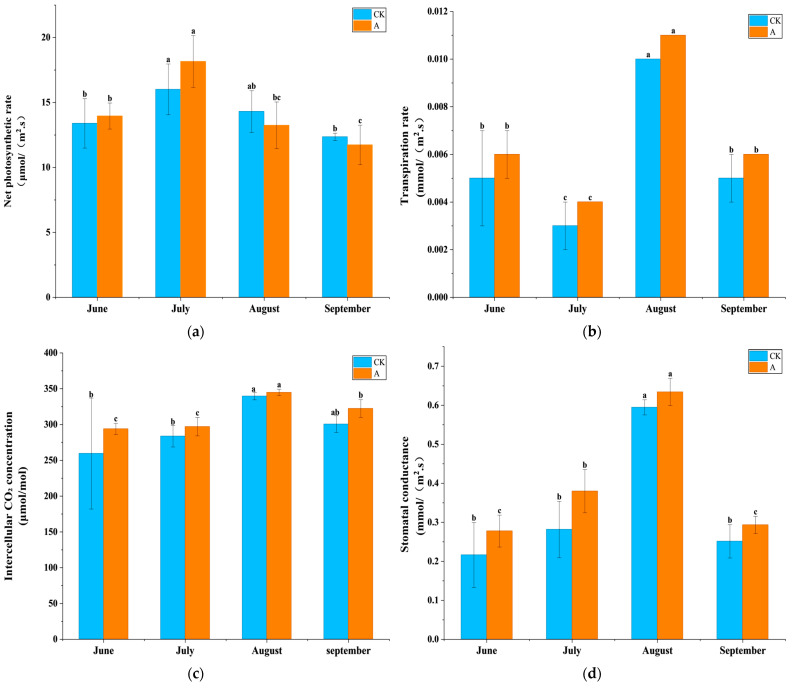
Effects of different treatments on apple photosynthesis in different months. Different lowercase letters in the same column indicate significant differences among treatments at *p* < 0.05, (**a**) Net photosynthetic rate; (**b**) transpiration rate; (**c**) intercellular CO_2_ concentration; (**d**) stomatal conductance.

Following *Morchella* intercropping, the photosynthetic performance of apple trees varied significantly across months (Figure 3). The net photosynthetic rate (Pn) in both treatments exhibited a unimodal pattern, increasing initially and then declining. In the *Morchella*-cultivated plots, Pn peaked in July at 18.16 μmol/(m^2^·s), which was significantly higher than in all other months. In the control plots, Pn also reached its maximum in July at 16.01 μmol/(m^2^·s), significantly exceeding the values recorded in June and September, though the difference from August was not statistically significant. Transpiration rate (Tr) in both treatments followed a pattern of initial decline, followed by an increase, and then a subsequent decrease. In August, Tr reached its peak at 0.011 mmol/(m^2^·s) in the treated plots and 0.010 mmol/(m^2^·s) in the controls, with both values being significantly higher than those recorded in all other months. Intercellular CO_2_ concentration (Ci) in both treatments also displayed a unimodal trend, increasing to a peak in August and then declining. In the treated plots, Ci in August (344.87 μmol/mol) was significantly higher than in all other months. In the control plots, the August value (339.68 μmol/mol) was significantly higher than those in June and July, but did not differ significantly from that in September. Stomatal conductance (Gs) in both treatments followed a similar unimodal pattern. In August, Gs values in the treated and control plots reached 0.63 and 0.60 mmol/(m^2^·s), respectively, both significantly higher than those in all other months. Collectively, these results indicate that, across the growing season, net photosynthetic rate peaked in July, while transpiration rate, intercellular CO_2_ concentration, and stomatal conductance all reached their maximum values in August, regardless of treatment.

### 3.6. Correlation Analysis Between Topsoil Physicochemical Properties and Soil Enzyme Activities Following Morchella Cultivation

**Figure 4:**
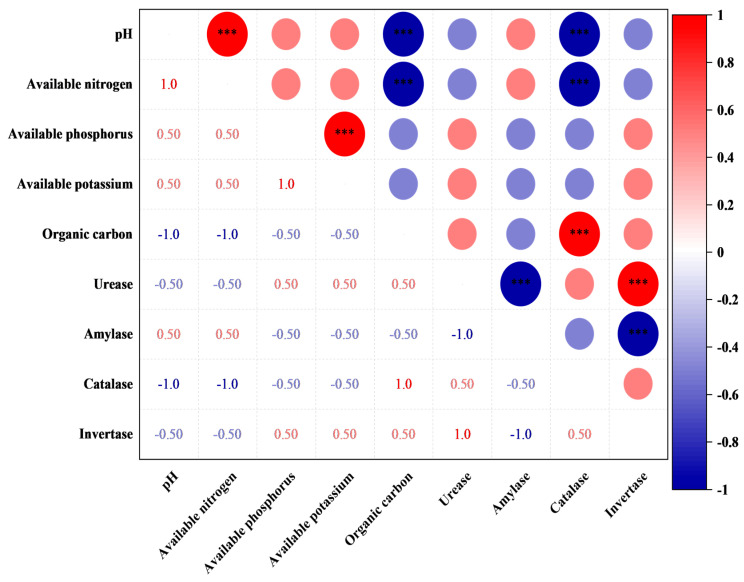
Correlation analysis of topsoil physicochemical parameters with soil enzyme activities after planting *Morchella*. *** indicate significant correlations at *p* < 0.001 level.

Following *Morchella* cultivation between apple rows, topsoil physicochemical properties and enzyme activities exhibited varying degrees of correlation (Figure 4). Specifically, soil pH showed a highly significant positive correlation with alkali-hydrolyzable nitrogen content, whereas both pH and alkali-hydrolyzable nitrogen were highly significantly negatively correlated with soil organic carbon content and catalase activity. Available phosphorus was highly significantly positively correlated with available potassium. Soil organic carbon content was highly significantly positively correlated with catalase activity. Urease activity showed a highly significant positive correlation with sucrase activity, but a highly significant negative correlation with amylase activity. Amylase activity was highly significantly negatively correlated with sucrase activity.

### 3.7. Correlation Analysis Between Topsoil Physicochemical Properties and Photosynthetic Performance of Apple Trees Following Morchella Cultivation

**Figure 5:**
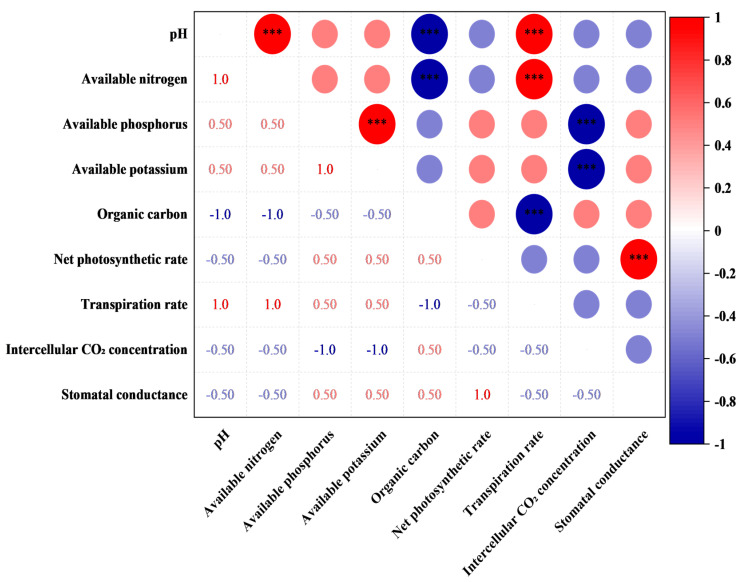
Correlation analysis between soil physicochemical properties and soil enzyme activities following *Morchella* cultivation. *** indicate significant correlations at *p* < 0.001 level.

Following *Morchella* cultivation between apple rows, topsoil physicochemical properties and photosynthetic parameters of the apple trees showed varying degrees of correlation (Figure 5). Specifically, soil pH exhibited a highly significant positive correlation with transpiration rate and a highly significant negative correlation with soil organic carbon content. Alkali-hydrolyzable nitrogen content was highly significantly positively correlated with transpiration rate, and highly significantly negatively correlated with soil organic carbon content. Available phosphorus was highly significantly positively correlated with available potassium, while both available phosphorus and available potassium were highly significantly negatively correlated with intercellular CO_2_ concentration. Soil organic carbon content was significantly negatively correlated with transpiration rate. Net photosynthetic rate (Pn) was highly significantly positively correlated with stomatal conductance (Gs).

## 4. Discussion

Apple fruit quality is closely associated with soil fertility, root development, and nutrient uptake capacity [25,26,27]. In this study, *Morchella* cultivation significantly increased individual fresh weight, as well as the contents of reducing sugars, total sugars, and soluble solids in apple fruits compared with the control. These improvements suggest that intercropping with *Morchella* enhanced soil physicochemical properties, creating favorable conditions for apple tree growth, which in turn increased fruit fresh weight and improved overall fruit quality. In return, the shaded and moist microclimate under the apple canopy provided suitable conditions for *Morchella* growth, thereby promoting the coordinated development of both apple trees and edible fungi. This mutually beneficial system contributes to the sustainability of agricultural production.

In this study, intercropping *Morchella* between apple tree rows significantly increased the natural soil water content in the 0–20 cm layer, suggesting that edible fungus cultivation can enhance soil water retention to some degree. This result is in agreement with earlier studies reporting increased soil moisture after cultivating *Coprinus comatus* under fruit trees [28] and edible fungi under *Cupressus* plantations [29]. This increase may be attributed to the substantial irrigation applied during *Morchella* cultivation, which directly raises soil water content [30]. In addition, as the fungal substrate is decomposed to support *Morchella* growth, it simultaneously remains on the soil surface, forming a physical mulch layer that reduces water evaporation and helps maintain soil moisture [31,32]. In this study, *Morchella* cultivation significantly increased soil pH, as well as the contents of available nitrogen and organic carbon in the 0–20 cm layer. This finding is consistent with the results of a previous study on the effects of spent mushroom substrate return on soil nutrient dynamics in paddy fields [33]. The observed increases in soil organic carbon and available nitrogen in the surface layer after *Morchella* cultivation likely result from a combination of factors. One key contribution comes from the residual substrate left in the nutrient packs, along with senescent mycelial debris, both of which act as exogenous organic inputs. These carbon- and nitrogen-enriched materials are gradually decomposed and mineralized by soil microbes, thereby releasing available nutrients into the soil [34,35].

Second, the large amount of mycelial biomass generated during *Morchella* cultivation, upon senescence and decomposition, deposits organic substances rich in chitin and proteins into the surface soil. Chitin, being a nitrogen-containing biopolymer, can gradually release nitrogen as it is broken down by microbial activity [36]. In addition, mycelial exudates and exogenous carbon sources may activate indigenous soil microorganisms, reinforcing the “microbial carbon pump” mechanism. This process facilitates the stabilization of organic carbon through the binding of microbial necromass to soil minerals [37,38], while simultaneously promoting the conversion of organic nitrogen to available nitrogen by ammonifying bacteria and related functional groups [39]. Nevertheless, further experimental validation is needed to elucidate the precise mechanisms involved. In the present study, a significant decrease in available potassium and organic carbon contents was observed in the 20–40 cm soil layer following *Morchella* cultivation. To interpret this unexpected finding, we put forward the following working hypotheses: First, *Morchella* mycelia possess strong penetrating ability, enabling them to extend into the 20–40 cm soil layer and continuously absorb available potassium to meet their growth requirements [40]. In addition, the 20–40 cm layer receives no exogenous organic matter inputs, leading to continuous depletion of the native carbon pool, which ultimately results in reduced organic carbon and available potassium contents. Second, the 20–40 cm layer is one of the primary zones of apple root distribution. The substantial uptake of nutrients, particularly potassium, by apple roots during the peak growing season may have exacerbated the decline in available potassium in this layer [41]. Furthermore, the improvement in soil structure and water status following *Morchella* cultivation may have enhanced root physiological activity, thereby increasing the trees’ capacity to acquire nutrients from deeper soil layers and indirectly contributing to the net nutrient depletion observed in the subsoil. However, the specific mechanisms underlying these effects require further investigation.

Soil enzyme activity is a key indicator of soil biological activity and fertility status [42]. Numerous studies have demonstrated that cultivating edible fungi under forest canopies can significantly activate the soil enzyme system, thereby promoting soil nutrient cycling and energy flow and ultimately enhancing soil fertility. This activation is primarily achieved through the stimulation of enzyme activities and soil microbial communities by metabolites secreted from fungal mycelia [43]. In the present study, enzyme activities generally decreased with increasing soil depth, which is consistent with the widely recognized surface-layer enrichment effect of soil enzyme activities [44,45]. The higher enzyme activities observed in the surface soil layer, compared with deeper layers, can be attributed to the more active exchange of materials and energy with the external environment at the surface, which favors microbial proliferation. With increasing soil depth, the declines in soil maturation degree, fertility level, and nutrient availability become less favorable for microbial activity and reproduction, ultimately resulting in reduced enzyme activities.

The magnitude of net photosynthetic rate (Pn) serves as a direct indicator of plant photosynthetic capacity. Transpiration rate (Tr) is an important physiological parameter for evaluating plant water metabolism and environmental adaptability. Stomatal conductance (Gs) is a key parameter reflecting leaf transpiration [46,47,48]. The results of this study showed that *Morchella* cultivation significantly increased the transpiration rate, intercellular CO_2_ concentration, and stomatal conductance of apple trees, with a pronounced promotional effect. To explore the possible mechanisms underlying this promotional effect, we propose the following hypotheses: On the one hand, the continuous respiratory release of CO_2_ from *Morchella* mycelia and fruiting bodies may increase the availability of photosynthetic substrate, thereby directly promoting photosynthetic carbon fixation. On the other hand, *Morchella* cultivation under the apple canopy may improve soil structure and activate soil nutrients, thereby enhancing plant water and nutrient uptake capacity, which in turn increases leaf stomatal conductance and light use efficiency [49]. In addition, the fungal layer under the forest canopy may help regulate the field microclimate, thereby mitigating environmental stress-induced damage to the photosynthetic apparatus. The synergistic effects of these multiple mechanisms may collectively contribute to the observed enhancement of photosynthetic performance, although the specific underlying causes require further investigation.

Changes in soil nutrient contents are closely associated with variations in soil enzyme activities. Intercropping *Morchella* beneath the apple canopy may have altered the patterns of root nutrient uptake and utilization, which in turn may be linked to the observed changes in soil enzyme activities. Correlation analyses between surface soil physicochemical properties and leaf photosynthetic parameters further revealed significant associations, suggesting that soil properties may profoundly influence apple photosynthetic capacity through multi-pathway cascading effects. However, the underlying causal mechanisms remain to be validated through controlled experimental approaches.

## 5. Conclusions

Intercropping *Morchella* between apple tree rows represents a novel cultivation system. This approach not only yielded high-quality *Morchella* with satisfactory production but also significantly improved the physicochemical properties of the 0–20 cm soil layer, as evidenced by marked increases in natural soil water content, pH, available nitrogen, organic carbon, and the activities of catalase and sucrase. In the 20–40 cm layer, however, significant reductions were observed in available potassium and organic carbon contents. In addition, *Morchella* cultivation increased individual fresh weight, fruit shape index, and the contents of reducing sugars, total sugars, and soluble solids in apple fruits, while also enhancing leaf gas exchange to some extent, as reflected by significant increases in transpiration rate, intercellular CO_2_ concentration, and stomatal conductance. The effects of understory edible fungus cultivation are multifaceted and depend on factors such as tree species and canopy density, which should guide the selection of suitable fungal varieties. This trial demonstrated the preliminary potential of *Morchella* cultivation under apple canopies; however, further randomized, multi-season studies are required to confirm these findings. Moreover, the long-term effects of edible fungus cultivation on soil physicochemical properties, microbial community structure, and tree growth represent a continuous process that warrants further in-depth investigation over extended time scales.

## Figures and Tables

**Table 1:** Nutrient bag formulation.

Material(s)	Weight
Wheat grain	134 g
Rice husk	32 g
Potassium dihydrogen phosphate (KH_2_PO_4_)	0.3 g
Gypsum (calcium sulfate)	3.8 g
Lime (calcium oxide/hydroxide)	2.8 g

**Table 2:** Comparison of yield and drying rate of *Morchella* across different planting sites.

Treatment	Yield g/m^2^	Drying Ratio %
P-YDJ	1333.74 ± 282.92 a	9.71 ± 0.82 b
L-YDJ	1118.88 ± 57.97 a	13.05 ± 0.19 a

Different lowercase letters within the same column indicate significant differences according to Fisher’s least significant difference (LSD) test (*p* < 0.05) and the same below. P-YDJ: interplanting *Morchella* between apple tree rows; L-YDJ: open-field cultivation of *Morchella*.

**Table 3:** Comparison of nutrient component contents in *Morchella* esculenta from different planting sites (%).

Treatment	Ash Content	Crude Fat	Crude Fiber	Total Sugar	Crude Protein	Reducing Sugar	Vitamin C	Free Amino Acids
P-YDJ	7.53 ± 0.24 b	6.58 ± 0.55 a	14.54 ± 0.24 a	19.38 ± 0.47 a	6.26 ± 0.25 b	27.65 ± 0.65 a	0.16 ± 0.00 b	0.78 ± 0.06 a
L-YDJ	7.79 ± 0.16 a	5.87 ± 0.63 a	12.69 ± 0.31 b	15.81 ± 0.23 b	7.42 ± 0.16 a	18.67 ± 0.70 b	0.28 ± 0.02 a	0.60 ± 0.03 b

Different lowercase letters within the same column indicate significant differences according to Fisher’s least significant difference (LSD) test (*p* < 0.05), and the same below. P-YDJ: interplanting Morchella between apple tree rows; L-YDJ: open-field cultivation of Morchella.

**Table 4:** Comparison of soil physical properties under different treatments.

Treatment	Natural Water Content (%)	Field Capacity (%)	Soil Bulk Density (g/cm^3^)	Total Porosity (g/cm^3^)	Capillary Porosity (g/cm^3^)	Non-Capillary Porosity (g/cm^3^)
CK_0_	13.86 ± 1.64 b	39.88 ± 5.70 a	1.52 ± 0.09 a	43.82 ± 2.93 a	0.52 ± 0.03 a	43.31 ± 2.89 a
A_0_	21.45 ± 0.27 a	44.09 ± 0.51 a	1.51 ± 0.01 a	44.07 ± 0.20 a	0.52 ± 0.01 a	43.55 ± 0.21 a

Different lowercase letters within the same column indicate significant differences according to Fisher’s least significant difference (LSD) test (*p* < 0.05), CK_0_: 0–20 cm soil without *Morchella* cultivation; A_0_: 0–20 cm soil with *Morchella* cultivation.

**Table 5:** Comparison of soil chemical properties under different treatments.

Treatment	pH Value	Alkali-Hydrolyzable Nitrogen (mg/kg)	Available Phosphorus (mg/kg)	Available Potassium (mg/kg)	Organic Carbon (g/kg)
CK_0_	5.33 ± 0.012 b	5.55 ± 0.49 b	6.31 ± 1.12 a	685.63 ± 11.52 a	30.33 ± 0.74 b
A_0_	5.46 ± 0.038 a	6.73 ± 0.17 a	5.25 ± 0.55 a	682.33 ± 7.51 a	38.30 ± 4.73 a
CK_1_	5.19 ± 0.038 a	6.15 ± 0.32 a	5.00 ± 0.58 a	570.07 ± 9.75 a	30.02 ± 0.85 a
A_1_	5.20 ± 0.035 a	5.71 ± 0.15 a	5.02 ± 1.03 a	418.37 ± 4.37 b	28.45 ± 0.48 b

Different lowercase letters within the same column indicate significant differences according to Fisher’s least significant difference (LSD) test (*p* < 0.05), and the same below. CK_0_: 0–20 cm soil without *Morchella* cultivation; A_0_: 0–20 cm soil with *Morchella* cultivation; CK_1_: 20–40 cm soil without *Morchella* cultivation; A_1_: 20–40 cm soil with *Morchella* cultivation.

**Table 6:** Comparison of soil enzyme activities under different treatments.

Treatment	Urease (μg/d/g)	Amylase (μg/h/g)	Catalase (μmol/h/g)	Sucrase (mg/d/g)
CK_0_	177.65 ± 55.23 a	1482.01 ± 46.55 a	369.55 ± 16.65 b	7.41 ± 0.56 b
A_0_	204.32 ± 77.87 a	1442.00 ± 78.47 a	411.86 ± 12.84 a	9.89 ± 0.93 a
CK_1_	173.47 ± 17.44 a	931.52 ± 253.16 a	392.57 ± 56.11 a	6.51 ± 0.25 a
A_1_	145.83 ± 29.43 a	947.49 ± 229.33 a	364.37 ± 32.60 a	7.03 ± 0.45 a

Different lowercase letters within the same column indicate significant differences according to Fisher’s least significant difference (LSD) test (*p* < 0.05) and the same below. CK_0_: 0–20 cm soil without *Morchella* cultivation; A_0_: 0–20 cm soil with *Morchella* cultivation; CK_1_: 20–40 cm soil without *Morchella* cultivation; A_1_: 20–40 cm soil with *Morchella* cultivation.

**Table 7:** Effect of different treatments on the nutrient content in apples.

Treatment	Single Fresh Weight (g)	Fruit Shape Index	Reducing Sugar (mg/g)	Total Sugar (mg/g)	Soluble Solids (%)	Titratable Acid (mg/g)	Vitamin C (mg/100g)	Fruit Firmness (N/cm^2^)
PG-CK	230.28 ± 22.93 b	0.81 ± 0.20 b	65.93 ± 3.99 b	94.38 ± 1.88 b	14.21 ± 0.31 b	0.25 ± 0.01 a	5.27 ± 0.06 a	179.41 ± 8.68 a
PG-A	262.29 ± 12.05 a	0.85 ± 0.03 a	82.53 ± 1.73 a	101.71 ± 3.09 a	15.81 ± 0.64 a	0.21 ± 0.02 a	5.15 ± 0.15 a	200.44 ± 19.71 a

Different lowercase letters within the same column indicate significant differences according to Fisher’s least significant difference (LSD) test (*p* < 0.05), PG-CK: apples from orchards without morel cultivation; PG-A: apples from orchards with morel cultivation.

**Table 8:** Effects of different treatments on photosynthesis in apple trees.

Month	Treatment	Net Photosynthetic Rate (μmol/(m^2^·s))	Transpiration Rate (mmol/(m^2^·s))	Intercellular CO_2_ Concentration (μmol/mol)	Stomatal Conductance (mmol/(m^2^·s))
June	CK_6_	13.30 ± 1.90 a	0.005 ± 0.002 a	292.62 ± 11.65 a	0.22 ± 0.08 a
	A_6_	13.96 ± 1.00 a	0.006 ± 0.001 a	293.88 ± 7.83 a	0.28 ± 0.04 a
July	CK_7_	16.01 ± 1.95 a	0.003 ± 0.001 b	283.79 ± 15.16 a	0.28 ± 0.07 b
	A_7_	18.16 ± 1.99 a	0.004 ± 0.000 a	297.02 ± 12.83 a	0.38 ± 0.06 a
August	CK_8_	14.31 ± 1.60 a	0.010 ± 0.000 b	339.68 ± 5.21 a	0.60 ± 0.02 a
	A_8_	13.24 ± 1.79 a	0.011 ± 0.000 a	344.87 ± 4.37 a	0.63 ± 0.04 a
September	CK_9_	12.35 ± 0.29 a	0.005 ± 0.001 b	300.60 ± 11.66 b	0.25 ± 0.04 a
	A_9_	11.73 ± 1.52 a	0.006 ± 0.000 a	322.38 ± 12.29 a	0.29 ± 0.02 a

Different lowercase letters within the same column indicate significant differences according to Fisher’s least significant difference (LSD) test (*p* < 0.05). CK_6_: apple trees from non-morel-cultivated plots measured in June; A_6_: apple trees from morel-cultivated plots measured in June; CK_7_: apple trees from non-morel-cultivated plots measured in July; A_7_: apple trees from morel-cultivated plots measured in July; CK_8_: apple trees from non-morel-cultivated plots measured in August; A_8_: apple trees from morel-cultivated plots measured in August; CK_9_: apple trees from non-morel-cultivated plots measured in September; A_9_: apple trees from morel-cultivated plots measured in September.

## Data Availability

The authors confirm that the data supporting the findings of this study are available within the article.

## Abbreviations

The following abbreviations are used in this manuscript:

P-YDJ	Interplanting *Morchella* between apple tree rows
L-YDJ	Open-field cultivation of *Morchella.*
CK_0_	0–20 cm soil without *Morchella* cultivation
A_0_	0–20 cm soil with *Morchella* Cultivation
CK_1_	20–40 cm soil without *Morchella* cultivation
A_1_	20–40 cm soil with *Morchella* cultivation
PG-CK	Apples from orchards without morel cultivation
PG-A	Apples from orchards with morel cultivation
CK_6_	Apple trees from non-morel-cultivated plots measured in June
A_6_	Apple trees from morel-cultivated plots measured in June
CK_7_	Apple trees from non-morel-cultivated plots measured in July
A_7_	Apple trees from morel-cultivated plots measured in July
CK_8_	Apple trees from non-morel-cultivated plots measured in August
A_8_	Apple trees from morel-cultivated plots measured in August
CK_9_	Apple trees from non-morel-cultivated plots measured in September
A_9_	Apple trees from morel-cultivated plots measured in September.
